# The impact of the absorbent products distribution system on family caregivers of older people with incontinence in Italy: perception of the support received

**DOI:** 10.1186/s12877-019-1254-4

**Published:** 2019-08-29

**Authors:** Sara Santini, Paolo Fabbietti, Giovanni Lamura

**Affiliations:** 1 IRCCS INRCA - National Institute of Health and Science on Ageing, Centre for Socio-Economic Research on Ageing, Via S. Margherita 5, 60124 Ancona, Italy; 2 Unit of Geriatric Pharmacoepidemiology, IRCCS INRCA - National Institute of Health and Science on Ageing, Contrada Muoio Piccolo, 87100 Cosenza, Italy

**Keywords:** Family caregivers, Older people with incontinence, Pads distribution, Regional health care system, Support, Services

## Abstract

**Background:**

Urinary incontinence is a chronic, age-related disorder, likely to increase in the future due to global population ageing. In Italy, as in most countries, older people with incontinence are often cared for by family caregivers, whose burden might be worsened by the perception of receiving an inadequate support, due to the lack of customized services. The aim of this study was to evaluate the impact of the absorbent products distribution method on family caregivers’ perception of the support received.

**Methods:**

The study compared the distribution of pads to homes and in pharmacy via a survey reaching 101 family caregivers of older people with incontinence living in two geographical areas of the Marche Region (Central Italy) with different distribution systems. The association between “Quality of perceived support” (the outcome variable) and two types of absorbent products delivery methods (i.e. pharmacy and home distribution) was analysed by means of a general linear model.

**Results:**

Findings show that family caregivers receiving pads at home (HODs) perceived a higher support than those gaining them at the pharmacy (PHADs) (respectively 68.1% vs 35%). The association between perceived support level and distribution system remained even after correction for confounding factors. 70.2% of PHADs reported “Poor well-being”, versus only 53.7% of HODs. The latter are more satisfied with the type of products distribution and thus less inclined to experiment different systems for the supply of products for the urinary continence (e.g. by voucher). The results are virtually reversed among PHADs and the difference is statistically significant (*p* <  0.001).

**Conclusions:**

When family caregivers feel supported by a more customized service delivery system, their perception of the care-related burden is mitigated. Thus, it is important to consider the needs of both family caregivers and cared for older people, and not only of the latter for designing a more suitable distribution of absorbent products. The best solution could be leaving end-users the freedom to choose how they want to get products (e.g. voucher or personal budget). This requires a reorganization of the current pads delivery systems adopted by the Marche and by other Italian Regional Health Systems.

## Background

According with the definition of Abrams et al. [1], which is accepted and adopted also by recent clinical and epidemiological studies [2, 3], urinary incontinence (UI) is the condition in which an involuntary loss of urine occurs, which is well identifiable and can create problems of a hygienic and social nature. Although the International Continence Society (ICS) withdrew the hygienic and social aspects of UI from the latest definition reported by the ICS Glossary, this study adopted the statement of meaning of Abrams et al. [1], because it is broader and multidisciplinary and so compliant with the purpose of this research, i.e. to look at the UI and at its repercussions on family caregivers of older people with incontinence from a sociological perspective. UI is often a chronic, age-related disorder, frequently occurring in association with disability [4, 5]. The unprecedented and worldwide population ageing implies, therefore, that the prevalence of this phenomenon is likely to increase in the future. In Italy, for example, where 22.3% of the whole population is over 65 years old [6], there are about 4,5 million people suffering from UI (i.e. 7.8% of the population) [7]. This condition concerns between 15 and 35% of older people living in the community, and 30% of those in nursing homes and facilities [7].

In Italy, as well as in Europe, older people with incontinence (OPI) are assisted mainly by family caregivers, often daughters, daughters-in-law and wives who deal with their older relatives’ personal hygiene, change of absorbent devices and frequent washing of linen. This is not surprising, as family caregivers are often the main source of assistance in the informal care sector, representing the backbone of long-term care provision across Europe [8–11].

Several studies highlight that the physical and psychological burden of family caregivers of OPI is higher than that experienced by family caregivers of older people without incontinence [12–15], and that such a burden can be mitigated by social and healthcare services meeting family caregivers’ needs [13]. Literature shows also that when caregivers of OPI feel that they are supported in providing assistance, their perception of the burden of care may be alleviated [16].

In Italy, services that explicitly target family caregivers are extremely limited, while those targeting OPI are more frequent. Among the latter, the main – and often only – service delivered by the Regional Health Systems (RHS) across the country is the free supply of absorbent products [17] through provincial administrative structures called *Aree Vaste* (literally “Large Areas”, occupying a position midway between the provincial and regional scale). The latter are responsible for the recognition of the disorder and of the granting of the free supply of absorbent products. In some regions, including the Marche region, each Large Area (LA) can decide for itself whether to distribute the products via pharmacies or deliver them to homes. As a result, methods of distribution of these products may differ within the same region. The number of absorbent products to be delivered each month and their type (model), in contrast, are decided by a regional law, which transposes and applies national legislation locally. For example, the Marche Region (Central Italy), where this study was carried out, at the time of this study data collection, allocated 60 pieces per month to individuals with incontinence, regardless of the severity of their disorder (Regional Law n. 1696/2012, which incorporated the law n. 135/2012, widely referred to as the “Spending Review”). Recently the Marche Region, in accordance with Law n. 716/2017, has doubled this amount, thus granting now 120 absorbent products per month (up to 150 for cases of serious incontinence), and delegating the LAs to decide the number of products to be granted. Other Italian Regional Health Systems (for example the Lombardy Region) have adopted the use of vouchers (i.e. a pre-paid receipt), but only as an experiment.

International literature has taken into consideration the impact of the quantity and quality of absorbent products on the well-being of people with incontinence and their caregivers [18–20], and has examined the cost of incontinence management in terms of public expenditure [21, 22]. In Italy, two studies [23, 24] highlighted the negative impact of complex procedures and lengthy waiting times to receive absorbent products in an informal care setting. To date, however, there are no studies at national as well as international level that have examined the impact of the method of distribution of these products on family caregivers’ perception of the support received.

This study, conducted in the Marche Region between January and July 2016, seeks to identify the method of distribution of absorbent products that provides Italian family caregivers of OPI with the greatest support, and whether this can mitigate their burden. The research question which the study tried to answer was: do caregivers who receive the products at home feel more supported than those who collect them at the pharmacy?

## Methods

### Study design and participants

To answer this research question, two groups of family caregivers, living in two LAs of the Marche region with two different systems for the supply of absorbent products were compared. The first group lived in Ancona (the region’s capital city) and collected the products via pharmacies. The second group lived in Fermo (a small town in the south of the region’s inland area), and received the products at home.

The sample size was estimated in order to obtain the representativeness of the family caregivers of OPI residing in Ancona and in Fermo. In 2015, the older population of Ancona had 25,659 subjects [25]. In the health district of Ancona in the same year, according to the data supplied by the Marche Region Health System, 1700 older people were entitled to receive absorbent aids, i.e. 7% of the total older population. Fermo counted 9251 older people [25]. However, for Fermo the number of residents who had applied to receive absorbent aids was not available. The estimation of the sample, therefore, was based on available data for the regional capital. Consequently, assuming a prevalence of urinary incontinence in the older male population of between 11 and 34%, and that of between 5 and 69% in the older female population [5], a sample of at least 100 subjects was estimated to be sufficiently representative of the OPI living in the Marche region. In order to ensure that the study complied with national ethical standards, the research protocol was submitted for approval to the Ethical Committee of the Marche Region, which deemed that ethical approval was not required as it was not a clinical trial.

An ad hoc questionnaire was administered to 101 family caregivers of OPI, who were included in the study on the basis of the following criteria: they had to be caregivers of older people with a medium-to-severe incontinence level (without a catheter), who used at least 2 pads daily, and who were residents in the municipalities of Ancona and Fermo.

Family caregivers were recruited from among all patients with UI admitted to the geriatric and neurology departments (which according to internal statistics had the highest annual OPI admissions) of two geriatric hospitals in Ancona and Fermo, between January and July 2016. Participants were recruited simultaneously in the two different geographical areas of the Marche region.

Family caregivers were informed by the head nurse about the aims of the study and that they might be interviewed. They were then contacted in the ward by interviewers and informed about the aims and methods of the study, in compliance with national personal data protection legislation (Law no. 196 of 2003, recently coordinated with the 2018 General Data Protection Regulation). Only family caregivers who signed the informed consent form and agreed to take part in the study were interviewed, i.e. 89.4% of the caregivers contacted.

### Measurements

The questionnaire consisted of questions relating to socio-demographic (age, gender, living and working conditions, etc.) and health-related factors concerning both family caregivers (self-assessment) and OPI, who were assessed according to the Instrumental Activities of Daily Living (IADL) scale [26–28]. The variables generated by the self-assessment questionnaire for family caregivers and the IADL scale for OPI were used to analyse the main characteristics of both counterparts and the management of household assistance overall. A variable, “IADL lost”, was built, indicating the sum of instrumental activities that the OPI in question can no longer perform. This variable was assigned a value of “<= 9”, if the older person was no longer able to perform 9 out of 10 activities, while it was assigned a value of “= 0” if he/she had lost the ability to perform all instrumental activities. The “chronic disease” variable is a binary (yes/no) variable. The variable “Number of absorbent products used in 24 hours” was broken down into three values: “<= 2” if the older person changed 1 or 2 pads a day, “3–5” for 3 to 5 changes, and “> 5” if the older person required more than 5 pads a day.

The outcome variable of the study is the “Quality of perceived support”, which was generated by the COPE Index “Quality of support” subscale, which has a reliability of α = 0.77–0.78 [29, 30].

Regarding the validity of this measurement, it is most highly correlated with the well-being and quality of life measures and with the Social Restriction Scale [30, 31]. The “Quality of support” subscale raw score ranged from 4 to 16. The score was dichotomised at a cut-off corresponding to the 50th percentile and was divided into two classes: a value between 4 and 10 indicated a low level of perceived support, while 11 and higher indicated a high level of perceived support.

The 5-Well-Being Index [32] is a short questionnaire used to assess the family caregivers’ subjective psychological well-being. The raw score ranges from 0 to 25, where 0 represents the worst possible and 25 the best possible quality of life. A score below 13 indicates poor wellbeing, which is an indicator for testing for depression and an appropriate tool for screening this disorder [33]. In light of this, the variable “poor wellbeing”, identifying family caregivers at risk of depression, was assigned the value “1″ if the index was < 13 or if at least one of the 5 items of the index had a score of 0 or 1. The variable was assigned the value “0″ if the index was > 13.

The variable indicating the opinion of caregivers on means of the pads distribution type they utilised, was followed by an open question to specify the reasons for the answer provided. Caregivers’ preferences on how they would like to receive absorbent aids were also taken into consideration through a multiple-choice question.

Finally, family caregivers were asked to give their opinion on the adoption of a voucher, i.e. a pre-paid receipt, which LAs would provide for people with incontinence for the free supply of absorbent products. The voucher-based system would allow users to choose their preferred type of absorbent products as long as the total cost of the products is below the expenditure threshold covered by the voucher. Respondents were asked to complete the following sentence: “The adoption of a voucher system for receiving absorbent products would be …” with one of the following options: “positive”, “indifferent”, “negative”, and were also asked to provide the reason for their choice through an open answer.

### Data analysis

Data are reported as mean (± SD) for continuous variables, and as absolute frequencies for categorical variables. Patients’ characteristics, divided by geographical area of residence, were compared using the chi-square test for categorical variables, and the t-Student test for continuous variables. A probability value of < 0.05 was considered statistically significant. Statistical analysis was performed using SPSS for Win V21.0 (SPSS Inc., Chicago, IL, USA). The association between “Quality of perceived support” and two geographical areas/methods of absorbent products delivery (i.e. Ancona and Fermo, respectively pharmacy distribution and home distribution) was analysed by means of a generalized linear model (GLM) by adjusting for the following confounding factors: family support (i.e. having a co-carer), income (i.e. being in employment and having any source of income) and living arrangements (i.e. cohabiting with the older person) [34, 35].

## Results

The 101 family caregivers of OPI enrolled in the study were asked to answer a questionnaire that included questions addressing both respondents and older people. The demographic characteristics of the sample of family caregivers and of the older relatives for whom they care are shown in Table 1. The mean age of the family caregivers is 60.5 years; 83 respondents are females and 82 people are married. Almost two thirds of respondents (65 people) perceive their health as “bad” or “very bad”. 51 respondents live with the assisted older person, in the same apartment, while 15 people live in the same building, but in different apartments. Only 8 family caregivers live 30 min away by car. 72 have secondary and higher education, while 12 have a bachelor’s degree. 39 family caregivers are in paid work (26 full-time and 13 part-time job), 50 do not work (retired and unemployed) and 11 women are housewives. 89 caregivers provide their loved ones with emotional and psychological support, 79 provide physical and practical support, and 76 help the older relative with personal hygiene (multiple answers). 80 respondents of the 101 carry out activities directly connected to incontinence (i.e. cleaning, changing of absorbent aids and washing of linen) on a daily basis or at least twice a week.

**Table 1:** Characteristics of family caregivers to care recipients

Family caregivers’ characteristics	All (*n* = 101)
*Age (years)*	60.5 (10.34)
*Gender*	
Male	19
Female	82
*Marital status*	
Unmarried	11
Married	82
Widower	4
Divorced/Separate	4
*Health condition self-evaluation*	
Very good/Good	10
Nor good neither bad	25
Bad/Very bad	65
*Living condition*	
With the cared for	51
With spouse, cared for and children	14
With cared for and migrant care worker	4
With spouse, cared for and migrant care worker	2
*Household proximity from the older person*	
Same apartment	51
In the same building but in different apartments	15
At walking distance	10
At 10 min by car bus or train	16
At 30 min by car bus or train	8
*Educational level*	
None	3
Primary School	13
Secondary School	36
High School	36
Bachelor degree	12
*Employment*	
Full-time employed	26
Part-time employed	13
Retired	43
Unemployed	7
Housewife	11
*Caregiving activities (from 3 times a week to every day)*	
Emotional, psychological and social support	89
Physical help	79
Medicine administration	78
Housework	77
Personal hygiene	76
*Frequency of caregiving activities related to the continence management*	
Rarely/never	20
Once/twice a week at least	80
Care recipients’ characteristics	
*Age (years)*	83.6 (9.35)
*Gender*	
Male	42
Female	59
*Number of pads per day*	
< 2	10
3–5	60
> 5	31
*IADL lost*	
< =9	45
10	56
*Chronic diseases*	
No	6
Yes	95

Data are mean (SD) or number of cases

The mean age of OPI is 83.6 years. 43 are men and 59 are women. 10 respondents use up to two diapers a day, 60 use between 2 and 5, and 31 use more than 5 per day. 45 OPI have lost up to 9 out of 10 of the activities in the IADL and 56 have lost 10 IADL. 95 out of 101 older people suffer from a chronic illness.

The sample was divided into two groups, according to the method of distribution of the absorbent products. The first group consisted of 47 caregivers (46.6% of the sample) who collected the continence products in pharmacies. We called “PHAD” (standing for “Pharmacy Distribution”). The second group consisted of 54 caregivers (53.4% of the sample) who received continence products at home, which we called “HOD” (for “Home Distribution”). Table 2 compares the main results for the two groups of caregivers.

**Table 2:** Family caregivers’ level of support perceived, wellbeing and opinion on the pads delivery per method of distribution

	Type of pads delivery system		P
	PHADs (*n* = 47)	HODs (*n* = 54)	
Family caregivers’ level of support perceived			
Low	26 (65.0)	15 (31.9)	0.002
High	14 (35.0)	32 (68.1)	
Poor well-being			
No	14 (29.8)	25 (46.3)	0.089
Yes	33 (70.2)	29 (53.7)	
Family caregivers’ opinion about the modality of distribution of pads			
"not suitable at all”	5 (11.3)	0 (0.0)	< 0.001
"acceptable”	25 (56.8)	5 (9.6)	
"completely suitable”	14 (31.8)	47 (90.4)	
Family caregivers’ preferencesconcerning the method f distribution of continence products			
At home	21 (47.7)	0 (0.0)	< 0.001
Pharmacy	17 (38.6)	0 (0.0)	
“It’s ok”	6 (13.6)	54 (100.0)	
Family caregivers’ opinion on voucher			
"It is positive”	35 (76.1)	23 (43.4)	< 0.001
"It does not make any difference”	11 (23.9)	20 (37.7)	
"It is negative”	0 (0)	10 (18.9)	

Data are number of cases (percentage)

Concerning the perceived support, 65% of caregivers in the PHAD group perceived a lower level of support (<= 10) and 35% perceived a higher level of support (> = 11). The results are virtually reversed among caregivers in the HOD group, 31.9% of whom perceived a lower level of support and 68.1% a higher level of support. The difference is statistically significant (*p* = 0.002).

The association between perceived support level and distribution method remained significant even after correction for family support, income and living solution, according to the GLM. In addition, 70.2% of caregivers in the PHAD group reported “Poor well-being”, as against only 53.7% in the HOD group.

Moreover, concerning the respondents’ opinions on the pads delivery method, 56.8% of caregivers in the PHAD group considered defined the distribution via pharmacies as “totally adequate”, 31.8% considered it “adequate”, and 11.3% “not at all adequate”. The main reason for the latter response was difficulty for caregivers in leaving the workplace or the older people to go to the pharmacy. The responses changed significantly among caregivers in the HOD group, 90.4% of whom found home delivery as “totally adequate”, 9.6% “adequate”, and none found it “not at all adequate”. Almost one half of caregivers in the PHAD group (47.7%) would prefer to receive products at home, while all caregivers in the HOD group were satisfied with home delivery and did not want to change.

76.1% of caregivers in the PHAD group were positive with regard to the introduction of a voucher system for the supply of absorbent products, as against only 43.4% of caregivers in the HOD group. 23.9% of caregivers in the PHAD group and 37.7% in the HOD group were indifferent. None in the PHAD group viewed the voucher system negatively, while 18.9% of those in the HOD group did. The difference is statistically significant (*p* <  0.001).

## Discussion

The aim of this study was to determine to what extent the method of distribution of absorbent products might affect the perception among OPI family caregivers of the support received and whether this, as a consequence, might influence the caregiving burden.

In light of our descriptive data analysis, the characteristics of family caregivers and OPI are consistent with what is reported in the literature. Concerning family caregivers, our data confirms the prevalence of women, the poor health conditions experienced, and the heavy care burden imposed by managing incontinence [8–15]. As far as OPI are concerned, our results underline the very advanced age, the reduced autonomy, and high incidence of chronic diseases.

In order to assess the impact of the method of distribution of absorbent products on the perception of support received among family caregivers of OPI, the study compared two groups of caregivers: the first collected the products from pharmacies (PHADs) and the second received them at home (HODs).

The results show that there is a significant association between the specific absorbent products distribution system and family caregivers’ perception of support received. Indeed, although PHADs and HODs received the same number of pads per month and of very similar quality, HODs perceived a greater level of support and reported higher levels of satisfaction with the service, which they did not wish to change.

Moreover, the difference in the percentages of respondents reporting “Poor well-being” in the two groups (70.2% of PHADs vs 53.7% of HODs), albeit not statistically significant, should lead to additional research on the possible association between these two items (i.e. pads distribution system and caregivers’ well-being). Indeed, people reporting “Poor well-being” rate might be at severe risk of depression [32].

The results, therefore, show that the distribution of absorbent products to the recipients’ homes better meets the needs of family caregivers involved in this study, making them feel better supported, with possible positive repercussions on their well-being.

This study sheds light on the effects of the method of distribution of absorbent products on the caregiving burden. In doing so, it fills a gap in the existing literature, which is focused mainly on the impact of the quantity and quality of the continence devices on the management of the continence care [18–20] and on the public expenditure [21, 22].

Although a number of confounding factors were taken into account in the analysis (i.e. family support, living arrangements and income) [34, 35], social and cultural patterns, e.g. representations of elderly care, traditions and values system [36], might have influenced the perception of the support received by family caregivers, which may be different in urban (i.e. Ancona) and rural (i.e. Fermo) areas. This study, due to its overall design, was not able to distinguish to what extent social and cultural factors might have influenced respondents’ opinions. It is therefore recommended that future research addresses this issue, by including qualitative questions to identify the cultural drivers of individuals’ preferences.

In this respect, a mixed-method approach [37, 38] seems to be the most appropriate means of data collection for appropriately dealing with this issue. Such an approach might help take into account the multifactorial nature of caring for an older person with incontinence (such as multimorbidity, the need for constant watchfulness, the physical effort and psychological stress of family caregivers and so on), as well as the sensitivity of this kind of care, as it is closely associated with the patient’s dignity and the risk of stigma and social exclusion. This study has only partially adopted this approach by adding some open questions. Nevertheless, in view of the above, it would be more appropriate for future studies to combine semi-structured interviews with the questionnaire.

Despite these limitations, this is the first study in Italy to examine the effects of the distribution of absorbent products on family caregivers’ perception of the support received, and its results could help to design more effective services for supporting them. The method of distribution of absorbent products is the meeting point between the citizen with a healthcare and the health service from which the response to such a need is expected. It should therefore be adapted to the needs of users, in order to ensure a more efficient match between health demand and supply, thus also improving family caregivers’ well-being.

## Conclusions

This study has analysed the level of support perceived by family caregivers of OPI in relation to the method of distribution of absorbent products, thus filling a gap in the literature available today. The results seem to suggest that the home distribution of absorbent products is of greater help for family caregivers of OPI living in the Marche region in Italy, regardless of working, housing and economic conditions. In light of these outcomes, any reform of the services supporting dependent OPI should therefore also take into account the preferences of their family caregivers concerning the method used to supply absorbent products, in order to provide a truly useful service. Such a reform should start from a systematic assessment of families’ health and social needs. The health conditions of care recipients and family caregivers change over time, as do the formal support networks (e.g. public and private long-term care services) and the informal support networks (e.g. family members and close friends) on which they can count. Therefore, Italy’s Large Areas and the Regional Health Systems should periodically distribute questionnaires to and conduct interviews with older people with incontinence and their family caregivers, in order to identify and monitor their needs and preferences not only regarding the quality and quantity of absorbent products (i.e. the “what” of the service), but also the method of distribution (i.e. the “how” of the service). Once the demand for health services has been assessed, the second step should be to trial new solutions for products distribution designed on the basis of the insights gathered, for example by doing a voucher-based system (where it is not yet in place) or a “personal budget”, i.e. an amount of money granted by the RHS which people can use to buy absorbent products wherever they want (for example from supermarkets) with the option of home delivery.

Indeed, given the enormous commitment required to family caregivers, any choice that saves them time and effort, including the method of distribution of absorbent products, can potentially mitigate the negative effects of care on their health and well-being. Allowing family caregivers to choose their preferred method of pad distribution would appear to be the best option in terms of the utility of the service. Just as the “one size does not fit all” principle was applied to the quality of absorbent products, it can similarly be said that “one pad distribution system does not fit all”.

## Data Availability

Data and material are available on request from the corresponding author.

## Abbreviations

GLM: General Linear Model
HOD: Home Distribution
IADL: Instrumental Activities of Daily Living
LA: Large Area
OPI: Older People with Incontinence
PHAD: Pharmacy Distribution
RHS: Regional Health System
